# Integrated GWAS and transcriptomic analysis reveal the candidate salt-responding genes regulating Na^+^/K^+^ balance in barley (*Hordeum vulgare* L.)

**DOI:** 10.3389/fpls.2022.1004477

**Published:** 2023-01-20

**Authors:** Tingting Xu, Shan Meng, Xiaopin Zhu, Jiachun Di, Yin Zhu, Xin Yang, Wei Yan

**Affiliations:** Institute of Germplasm Resources and Biotechnology, Jiangsu Key Laboratory for Agrobiolog, Jiangsu Provincial Platform for Conservation and Utilization of Agricultural Germplasm, Jiangsu Academy of Agricultural Sciences, Nanjing, China

**Keywords:** barley, GWAS, RNA-Seq, salt tolerance, Na +/K + balance, candidate genes

## Abstract

Salt stress is one of the main abiotic stresses affecting crop yield and quality. Barley has strong salt tolerance, however, the underlying genetic basis is not fully clear, especially in the seedling stage. This study examined the ionic changes in barley core germplasms under the control and salt conditions. Genome-wide association study (GWAS) analysis revealed 54 significant SNPs from a pool of 25,342 SNPs distributed in 7 chromosomes (Chr) of the Illumina Barley 50K SNP array. These SNPs are associated with ion homeostasis traits, sodium (Na^+^) and potassium (K^+^) content, and Na^+^/K^+^ ratio representing five genomic regions on Chr 2, 4, 5, 6, and 7 in the leaves of worldwide barley accessions. And there are 3 SNP peaks located on the Chr 4, 6, and 7, which could be the “hot spots” regions for mining and identifying candidate genes for salt tolerance. Furthermore, 616 unique candidate genes were screened surrounding the significant SNPs, which are associated with transport proteins, protein kinases, binding proteins, and other proteins of unknown function. Meanwhile, transcriptomic analysis (RNA-Seq) was carried out to compare the salt-tolerant (CM72) and salt-sensitive (Gairdner) genotypes subjected to salt stress. And there was a greater accumulation of differentially expressed genes(DEGs) in Gairdner compared to CM72, mainly enriched in metabolic pathway, biosynthesis of secondary metabolites, photosynthesis, signal transduction,emphasizing the different transcriptional response in both genotypes following salt exposure. Combined GWAS and RNA-Seq analysis revealed 5 promising salt-responding genes (PGK2, BASS3, SINAT2, AQP, and SYT3) from the hot spot regions, which were verified between the salt-tolerant and salt-sensitive varieties by qRT-PCR. In all, these results provide candidate SNPs and genes responsible for salinity responding in barley, and a new idea for studying such genetic basis in similar crops.

## 1 Introduction

With increasing soil salinization, the area of arable land is decreasing worldwide threatening crop production and food safety (Han et al., 2015; Ramamoorthy et al., 2017). Though the use of modern farming and irrigation techniques can alleviate soil salinization to some extent, these methods require high input and have a slow response. Therefore, screening or cultivating new varieties of salt-tolerant crops can fundamentally solve the problem of saline-alkali land improving crop yield and quality.

Excessive accumulation of Na^+^ in plant tissues, on the one hand, causes osmotic stress, making it more difficult for the root system to absorb water, resulting in accelerated leaf senescence and death. On the other hand, it leads to a serious imbalance of intracellular ions, which affects the absorption of nutrients such as K, N, P, Ca, Mg, and Fe, resulting in plant physiology and metabolic disorders (Ruiz et al., 1997). Especially, since Na^+^ and K^+^ have similar hydration radii, non-selective cation channels or transporters cannot distinguish them, resulting in a large amount of Na+ competitively inhibiting K^+^ uptake (Assaha et al., 2017), disturbing the intracellular Na^+^/K^+^ ratio, leading to programmed cell death (PCD) (Shabala et al., 2007), indicating that the main toxic ions in saline-alkali soil cause osmotic stress and ion toxicity in plant cells, trigger oxidative stress, and affect mineral nutrient metabolism, plant seed germination, crop yield, and quality (Munns and Tester, 2008; Janz and Polle, 2012; Zhu et al., 2017). To counter salt stress, plants have evolved several physiological and biochemical strategies (Slama et al., 2015), such as the regionalization of Na^+^ in vacuoles by Na^+^/H^+^ antiporter (NHXs) (Jiang et al., 2010; Zhang et al., 2018; Sun et al., 2019) and efflux of Na^+^ from the cytosol to the apoplast via the Salt Overly Sensitive (SOS) signal pathway (Yang et al., 2009; Nutan et al., 2018; Zhang et al., 2018). High-affinity potassium transporter (HKT), which regulates the Na^+^/K^+^ transport, reduces Na^+^ accumulation in leaves by regulating phloem loading of Na^+^ (Hamamoto et al., 2015; Suzuki et al., 2016; Kobayashi et al., 2017; Huang et al., 2020). In addition, plants can reduce salt stress-induced osmotic stress by accumulating osmotic protective substances such as proline, betaine, late embryogenesis-enriched protein (LEA), and carbohydrates (Cuin and Shabala, 2005; Cuin and Shabala, 2007; Jia et al., 2020). In addition, phytohormones (Verma et al., 2016), NO (Nitric oxide) (Yemets et al., 2011), and polyamines (Shi and Chan, 2014) play important roles in maintaining ion homeostasis and signaling in plant salt tolerance networks.

Barley (Hordeum vulgare L.) is an ancient cereal crop considered to be the world’s most tolerant crop to salt, drought, low temperature, and cold stresses, which can be used as a model crop for studying the molecular regulation mechanism of salt tolerance (Nevo and Chen, 2010). At present, a lot of quantitative trait loci (QTLs) of physiological and biochemical indicators under salt stress have been identified using bi-parental populations (Mano and Takeda, 1997; Huang et al., 2009; Shavrukov et al., 2010; Xue et al., 2017). However, QTL mapping is a time-consuming and laborious process, resulting in the limited elucidation of the genetic mechanisms of plant salt tolerance.

In contrast, genome-wide association studies (GWAS) can find SNPs positioned across the genome as molecular markers and, therefore, is an efficient and convenient tool to dissect the genetic basis of complex traits in the plant, such as Arabidopsis (Arabidopsis thaliana), rice (Oryza sativa), maize (Zea mays), wheat (Triticum aestivum), and cotton (*Gossypium hirsutum* L.) (Atwell et al., 2010; Chen et al., 2015; Fang et al., 2017; Sun Z. et al., 2018). In barley, the major salt tolerance controlling QTL is on Chr 6, and a strong QTL for ion content is on Chr 4, which were identified using 192 spring barley genotypes with 1,000 SNPs (Long et al., 2013). Sbei et al. (2014) identified 7 significant QTLs related to salt tolerance using 296 Asian barley and 384 SNPs. Recently, using the Illumina 9K SNP array (Comadran et al., 2012), HKT1;5 was mapped on the distal part of Chr 4, which negatively regulates the salt tolerance in barley (Hazzouri et al., 2018; van Bezouw et al., 2019; Huang et al., 2020). Mwando et al. (2020) identified 19 loci containing 52 significant salt-tolerance-associated markers for salinity tolerance during germination. In recent years, with the publication of the whole genome and pan-genome of barley (Mayer et al., 2012; Mascher et al., 2017; Jayakodi et al., 2020), the first commercial high-density Barley SNP50K array was developed recently (Bayer et al., 2017), which can be used to accelerate the mining of salt tolerance loci or genes that were used in crop breeding for breeders.

Accordingly, this study identified the natural allelic variation in the barley genome and candidate genes that are significantly associated with salt tolerance. Here, we performed a GWAS using a natural population of 288 barley core collections and the Illumina Barley 50K SNP array based on ion traits for salt tolerance. In addition, we performed transcriptome analysis and qRT-PCR to verify the candidate genes identified by GWAS. In total, we identified 54 SNPs and 5 putative candidate genes associated with Na^+^/K^+^ balance in barley leaves. The results might provide useful candidate SNPs and genes for high-quality barley salt-tolerance breeding and for understanding the mechanisms for salt tolerance in barley.

## 2 Materials and methods

### 2.1 Barley germplasm

To evaluate salinity tolerance, a natural population consisting of a global barley core collection of 288 barley accessions (149 two-rowed and 139 six-rowed) collected from different countries or regions was used in this study. Among the accessions, 129 were from different provinces in China, 156 were from Australia, Germany, Japan, and other countries, and 3 were from unknown geographical sources (**Supplementary Table 1**) stored in the Jiangsu Provincial Platform for Conservation and Utilization of Agricultural Germplasm.

### 2.2 Seedlings hydroponic culture

The experiment was carried out in the greenhouse at Jiangsu Academy of Agricultural Science, Nanjing, China. Seeds with full and non-destructive granules were selected, and germinated in a plug with one seed per hole at 22/18 °C day/night conditions. Later, the seedlings (at the 1.5-2.0 leaf stage; 7-10 days old) were transferred to a turnover box containing modified Hoagland’s nutrient solution as described by Qiu et al. (2011). The box was covered with a plastic lid with evenly spaced holes; each hole contained 2 seedlings bundled with foam. After 10 days of transplanting, NaCl was added to the nutrient solution by 100 mM NaCl per day to reach a final concentration of 300 mM NaCl. Finally, two treatments were produced: (1) control treatment: 0 mM NaCl. (2) salt treatment: 300 mM NaCl. The nutrient solution was continuously oxygenated and changed weekly. After 3 weeks of salt stress and control treatment, the barley samples were taken for measurement. The whole experiment used a completely random design (CRD) under factorial arrangement with 3 replicates and four plants per replication.

### 2.3 Evaluation of salt tolerance

To estimate salt tolerance in barley, data of seedlings phenotypes were collected and statistically analyzed after 3 weeks of different treatment conditions. After 3 weeks of salt treatment, the sampled seedlings were rinsed with deionized water and dried with absorbent papers. Immediately after sample collection, plant height, fresh and dry weights, and other phenotypes were measured. Na^+^ and K^+^ contents in dried leaves were determined using an ICP-OES/MS spectrometer as described by Qiu et al. (2011). Salt tolerance (ST) was estimated as treatment/control×100% based on Na^+^ content, K^+^content, and Na^+^/K^+^ ratio. Statistical analysis, Pearson correlation between traits, ANOVA, and principal component analysis were performed by SPSS 20 software.

### 2.4 Genotyping and screening of SNP markers

The genomic DNA was extracted from young leaf tissues for genotyping using the CTAB method (Attitalla, 2011). The barley germplasms were genotyped using the Illumina BarleySNP50K array containing 44,040 SNPs (Bayer et al., 2017). The SNP data was filtered and processed by plink 1.90, with the criteria of calling rate < 0.9 and MAF < 0.05 (Chang et al., 2015).

### 2.5 Population structure analysis

The genetic structure of the population was estimated using the STRUCTURE 2.3.4 software; the K value was set from 2 to 10 and 15 iterations were used in an admixture model (Evanno et al., 2005). The obtained results were uploaded to the STRUCTURE Harvester website (http://taylor0.biology.ucla.Edu/structureHarvester/), when △K achieved maximum value, the optimal K was the number of clusters. Principal component analysis (PCA) was used to assess the population structure by GAPIT software (Tang et al., 2016). A neighbor-joining (NJ) phylogenetic tree was constructed by TASSEL software using individual sequences.

### 2.6 Genome-wide association analysis

The LD parameter (*r^2^*) between pairwise SNPs (MAF > 0.05) was estimated using the PopLDdecay software (Zhang et al., 2019). The association analysis was performed using the genome association and prediction integrated tool package (GAPIT). The significantly associated SNPs for the target traits were identified by the criteria -log_10_ (*P*) ≥-log_10_ (1/*n*), where *n* denotes the total filtered SNPs.

### 2.7 Identification of candidate genes

Candidate genes located within and/or adjacent to the associated SNPs were identified through the Barleymap (http://floresta.eead.csic.es/barleymap/) and Gramene (http://ensembl.gramene.org/Hordeum_vulgare/Info/Index) using the Morex genome. GO (Gene Ontology) and KEGG PATHWAY enrichment analyses were performed through the GENE ONTOLOGY (http://geneontology.org/).

### 2.8 Transcriptome sequencing

The seeds of salt-tolerant barley CM72 (C) and salt-sensitive barley Gairdner (G) were grown in a greenhouse, in Nanjing, China. Seedlings were collected after 0h, 3h, 12h, and 48 h with three biological replicates under 300 mM salt concentration and frozen in liquid nitrogen. Total RNA was extracted from the leaf samples using the EASY spin Plant RNA kit (Aidlab, Beijing, China), and sequencing libraries were generated using NEBNextR UltraTM Directional RNA Library Prep Kit for IlluminaR (NEB, USA) following manufacturer’s recommendations. The libraries were sequenced on an Illumina Hiseq Xten platform and paired-end reads were generated. Raw data (raw reads) of fastq format were firstly processed through in-house perl scripts by removing reads containing adapter, reads containing ploy-N and low quality reads from raw data. Then the clean reads were mapped to the reference genome sequence (https://webblast.ipk-gatersleben.de/barley_ibsc/downloads/) by HISAT2 tools software. Only reads with a perfect match or one mismatch were further analyzed and annotated based on the reference genome. All the downstream analyses were based on clean data with high quality. The original datas of the transcription sequencing were submitted to SRA database at NCBI (accession number: PRJNA866193 and PRJNA892763).

Gene expression levels were estimated by fragments per kilobase of transcript per million fragments mapped (FPKM). The false discovery rate (FDR) < 0.05 and |log_2_ (foldchange (FC))| ≥1 were set as the threshold by DESeq2 softwore (Tu et al., 2021) for significantly differential expression to identify differentially expressed gene (DEG) in response to salt stress. GO enrichment analysis of the DEGs was implemented by the clusterProfiler R package. We used KOBAS (Mao et al., 2005) software to test the statistical enrichment of differential expression genes in KEGG pathways and the clusterProfiler R packages to find KEGG pathway that are significantly enriched compared to the entire genome background.

### 2.9 qRT-PCR of the putative candidate genes

Six salt-tolerant and six salt-sensitive varieties were selected and grown in the greenhouse for 48 h under 300 mM salt stress, among them, CM72 and Gairdner were used as reference varieties (**Supplementary Table 8**). Three biological replicates of seedlings were collected and frozen in liquid nitrogen for RNA extraction. Next, total cDNA was synthesized using the PrimeScriptTM RT reagent Kit (TaKaRa).

qRT-PCRs were performed in a 20 ml reaction system for 1 min at 95 °C, followed by 40 cycles at 95 °C for 15 s, 60 °C for 15 s, and 72 °C for 45 s: the reaction mixture included 2.0 μL of cDNA, 0.6 μL of primer, 10 μL of SYBR Premix DimerEraser (TaKaRa) and 6.8 μL ddH_2_O. The used primers are listed in **Supplementary Table 9**. *HvUBQ* was used as the reference gene. All reactions were performed using the ABI7500 Real-Time PCR System with three independent biological replicates.

## 3 Results

### 3.1 Phenotypic evaluation of salt-tolerance traits of barley at the seedling stage under contrasting growth conditions

**Figure 1:**
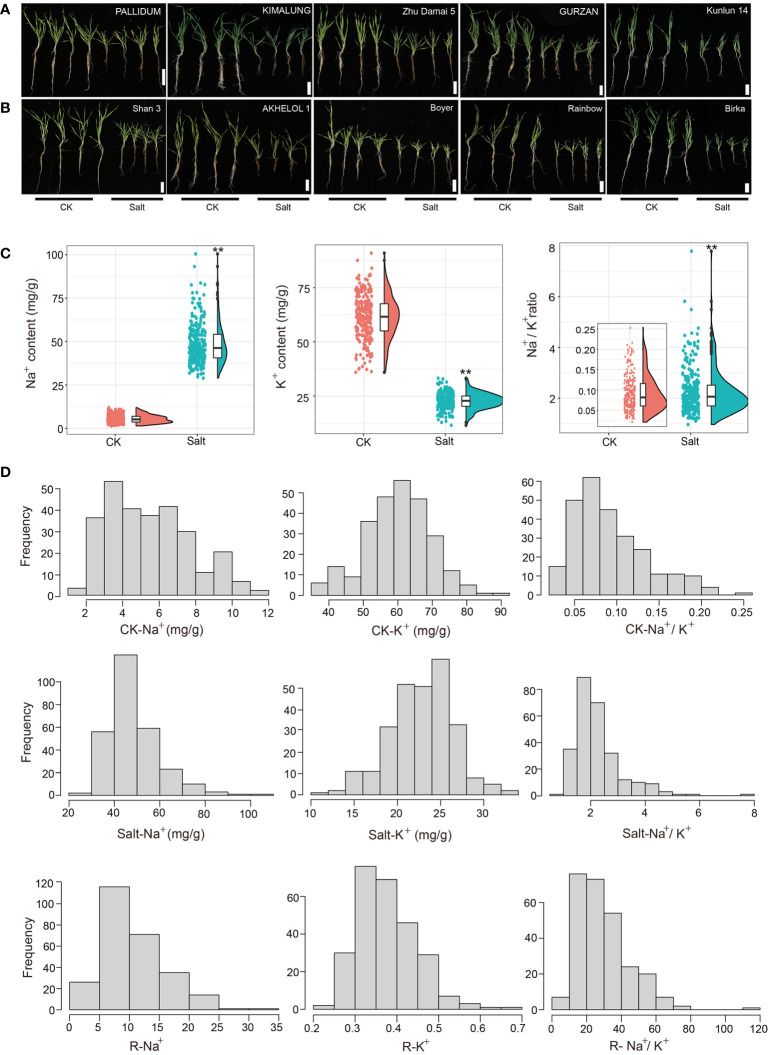
Phenotypic analysis of barley accessions under control (CK: 0 mM) and salt treatment (300 mM NaCl) conditions after 3 weeks. Seedlings growth performance of **(A)** salt-tolerant and **(B)** salt-sensitive varieties of barley. Bars, 10 cm. **(C)** Raincloud plots for Na^+^ content, K^+^ content, and Na^+^/K^+^ ratio traits under contrasting growth conditions. **p < 0.01 **(D)** Frequency distribution of Na^+^, K^+^content, and Na^+^/K^+^ ratio under contrasting growth conditions and relative value of Na^+^, K^+^, Na^+^/K^+^ ratio. CK: 0 mM NaCl, Salt: 300 mM NaCl.

To find the new salt-tolerant variety of barley, a set of 288 barley germplasms from all over the world were examined for salt tolerance at the seedling stage. After salt treatment (300 mM NaCl) for 3 weeks, the barley genotypes showed typical symptoms of salt damage, such as stunting, leaf wilting, and yellowing (**Supplementary Figure 1**; **Figures 1A, B**).

At the seedling stage, salt stress can affect the content of Na^+^ and K^+^ disturbing the ion balance, which affects the normal growth and development of plants. Compared with the control conditions, salt stress significantly increased the Na^+^ content (Salt-Na^+^) and Na^+^/K^+^ ratio (Salt-Na^+^/K^+^) from 5.46 to 48.66 mg/g (7.92-fold) and 0.09 to 2.25 (23.52-fold), respectively. Meanwhile, K^+^ content (Salt-K^+^) decreased significantly from 61.36 to 22.75 mg/g showing about a 62.92% reduction (**Figure 1C** and **Table 1**). Moreover, the relative value of Na^+^ (R-Na^+^), K^+^ (R-K^+^), and Na^+^/K^+^ (R-Na^+^/K^+^), which can be used to evaluate salt tolerance, ranged from 2.95 to 33.13, 0.21 to 0.66, and 6.42 to 115.75, respectively (**Table 1**), with a continuous distribution (**Figure 1D**), suggesting that the ion balance were controlled by multi-QTLs. The variance analysis based on ion-related traits showed significant differences among genotypes (**Supplementary Table 2**). Correlation analysis showed that Na^+^ and Na^+^/K^+^ were significantly negatively correlated with morphological traits (except YLN) and a positive correlation was also found between K^+^ and morphological traits (except YLN) (**Supplementary Table 3**). These results showed that salt stress conditions significantly increased the content of Na^+^ and reduced the content of K^+^, which disturbed the ion balance in barley and impaired the growth and development of plants.

**Table 1:** Phenotypic variation for the salt-tolerance traits under salt treatment in the panel of barley accessions.

Traits	Min	Max	Mean	SD	CV (%)	Skewness	Kurtosis	% of reduction
CK-Na^+^	1.41	11.86	5.46	2.26	41.48	0.50	-0.47	-791.96
Salt-Na^+^	28.95	100.43	48.66	11.56	23.77	1.24	2.14	
R-Na^+^	2.95	33.13	10.64	5.17	48.54	1.06	1.18	
CK-K^+^	35.95	90.88	61.36	9.93	16.18	-0.14	0.06	62.92
Salt-K^+^	11.54	33.22	22.75	3.65	16.06	-0.12	0.28	
R-K^+^	0.21	0.66	0.38	0.07	18.64	0.83	1.06	
CK-Na^+^/K^+^	0.02	0.25	0.09	0.04	47.30	1.00	0.67	-2352.25
Salt-Na^+^/K^+^	0.96	7.79	2.25	0.88	39.12	2.06	7.09	
R-Na^+^/K^+^	6.42	115.75	29.18	15.12	51.83	1.31	3.47	

CK-Na^+^, Na^+^ content in control treatment (CK; 0 mM); CK-K^+^, K^+^ content in control treatment (CK; 0 mM); CK-Na^+^/K^+^, Na^+^/K^+^ ratio in control treatment (CK; 0 mM); Salt-Na^+^, Na^+^ content in salt treatment (300 mM NaCl); Salt-K^+^, K^+^ content in salt treatment (300 mM NaCl); Salt-Na^+^/K^+^, Na^+^/K^+^ ratio in salt treatment (300 mM NaCl); R-Na^+^, Relative value of Na^+^; R-K^+^, Relative value of K^+^; R-Na^+^/K^+^, Relative value of Na^+^/K^+^ ratio.

We further identified salt-tolerant (**Figure 1B**) and salt-sensitive barley germplasm (**Figure 1B**) based on the value of Salt-Na^+^, Salt-Na^+^/K^+^, R-Na^+^, and R-Na^+^/K^+^ as indicators of salt tolerance, which was obtained by PCA analysis (**Table 2**), indicating that these could be valuable germplasm for the development of salt-tolerant cultivars.

**Table 2:** Principal component analysis of ionic traits.

Traits	PC1	PC2	PC3
CK-Na^+^	-0.713	0.608	-0.225
CK-K^+^	-0.082	-0.467	-0.833
CK-Na^+^/K^+^	-0.609	0.758	0.123
Salt-Na^+^	0.568	0.633	0.018
Salt-K^+^	-0.461	-0.718	0.11
Salt-Na^+^/K^+^	0.585	0.782	-0.025
R-Na^+^	0.896	-0.264	0.277
R-K^+^	-0.356	-0.214	0.886
R-Na^+^/K^+^	0.963	-0.135	-0.033

### 3.2 Genotyping and screening of SNPs

**Figure 2:**
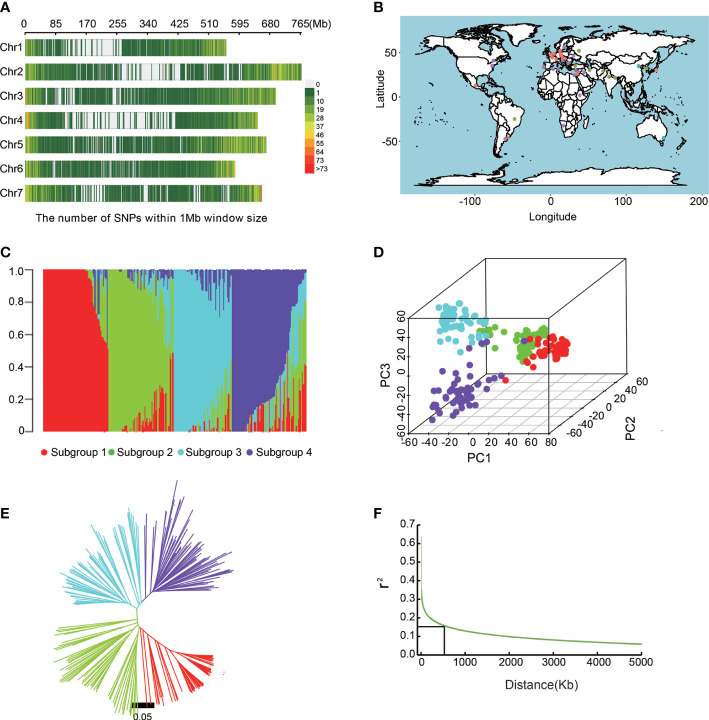
Population structure analysis of barley accessions. **(A)** Genome-wide SNP density in the entire association mapping panel. **(B)** Geographical distribution of barley accessions. **(C)** Population structure analysis revealed that barley accessions belong to four subgroups (K = 4). **(D)** Principal component analysis showing the four subpopulations. **(E)** A neighboring joining tree shows the four different subpopulations. **(F)** Genome-wide average LD decay distance in the barley genome.

The barley accessions were genotyped using the Barley 50K Illumina SNP array. A set of 25,342 mapped and high-quality SNPs were obtained using the criteria of MAF >0.05 and call rate >0.9 for genetic variation and GWAS analysis. These SNPs are evenly distributed on 7 chromosomes of barley (**Figure 2A**); Chr 5 has the maximum SNPs (4,707) while Chr 1 has the minimum (2,780). The polymorphism information content (PIC) values ranged from 0.354 to 0.369 among the 7 chromosomes (**Table 3**). At the same time, the T/C allele SNPs are the maximum (8,422), and the T/A allele SNPs are the minimum (1,656). This is consistent with the fact that each chromosome has the maximum number of T/C, while T/A is the least (**Supplementary Table 4**).

**Table 3:** The summary of the number of polymorphic SNPs mapped in barley genome.

Chr	No. of SNPs	Chr. Size (Mb)	Density of SNP (kb/SNP)	PIC
Chr1	2,780	558.35	200.84	0.360
Chr2	4,290	767.93	179.00	0.355
Chr3	4,038	696.52	172.49	0.369
Chr4	2,994	646.18	215.82	0.363
Chr5	4,607	669.37	145.29	0.354
Chr6	3,027	583.02	192.61	0.366
Chr7	3,606	656.80	182.14	0.357
Sum	25,342	4578.16		

Chr, chromosome; PIC, polymorphism information content.

### 3.3 Population structure and linkage disequilibrium analysis

We investigated the population structure of the barley germplasms based on 25,342 SNPs by STRUCTURE 2.3.4 software. At K = 4, the barley varieties were divided into 4 sub-populations (**Figures 2B, C** **;** **Supplementary Table 1**). Subgroup1 contained 74 barley accessions, about 70% of which were from abroad. Subgroup2 contained 77 barley accessions, of which, most were from Qinghai and Tibet. Subgroup3 contained 59 barley accessions mainly from China (44) and Japan (11), including 15 from Jiangsu. 78 barley accessions of subgroup4 were from Australia, North America, and Europe. Furthermore, the six-rowed barley belonged to subgroup2 and 4, while the two-rowed barley belonged to subgroup1 and 3. Meanwhile, PCA analysis (**Figure 2D**) and NJ tree also showed similar clustering results (**Figure 2E**).

We then analyzed LD in barley germplasm using filtered SNPs, and LD decay rates provided a moderate resolution for the identification of candidate gene regions (Bulik-Sullivan et al., 2015; Wijmenga, 2021). In this study, when the *r^2^* value drops to half of the maximum value, the LD decay distance was approximately 5Kb, showing that the natural population had high genetic diversity. However, considering the large size of the barley genome, and the previously reported LD decay distance of barley was 2-8M (Long et al., 2013), we fixed 500 Kb as an interval distance for screening candidate genes around significantly associated SNPs (**Figure 2F**).

### 3.4 Identification of significant SNP loci by GWAS

**Figure 3:**
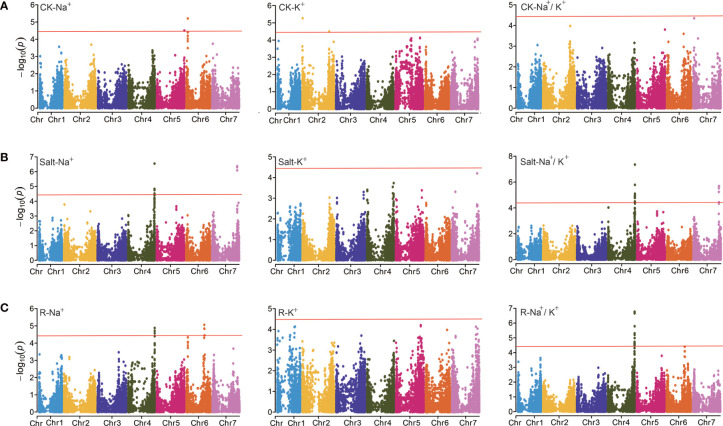
GWAS analysis for the ion-related traits under different conditions. **(A)** Manhattan plot for CK-Na^+^, CK-K^+^, and CK-Na^+^/K^+^, respectively. **(B)** Manhattan plot for Salt-Na^+^, Salt-K^+^, and Salt-Na^+^/K^+^, respectively. **(C)** Manhattan plot for R-Na^+^, R-K^+^, and R-Na^+^/K^+^, respectively.

To explore the genetic basis of salt tolerance in barley, 288 barley accessions, and 25,342 SNP markers were used for GWAS. In total, 54 significantly associated SNPs were found (criteria -Log_10_(*P*) ≥ 4.40) located mainly on Chr 2, 4, 5, 6, and 7. Some of these were detected repeatedly, indicating their true association with traits (**Figure 3** and **Table 4**).

**Table 4:** The summary of SNPs significantly associated with ion-related traits.

Traits	SNPs	Chr.	Site	Allele	MAF	-Log_10_ (*P*)	*R2* (%)
CK-K^+^	BOPA2_12_21415	Chr2	6193157	G/T	0.13	5.27	0.17
	JHI-Hv50k-2016-106402	Chr2	647542686	T/C	0.50	4.50	0.16
CK-Na^+^	JHI-Hv50k-2016-366579	Chr5	667544188	T/C	0.14	4.51	0.32
	JHI-Hv50k-2016-366592	Chr5	667546838	T/G	0.14	4.51	0.32
	JHI-Hv50k-2016-382879	Chr6	35313426	T/A	0.07	4.41	0.32
	JHI-Hv50k-2016-382988	Chr6	35396724	T/C	0.14	4.40	0.32
	JHI-Hv50k-2016-383140	Chr6	35794550	A/T	0.14	4.40	0.32
	JHI-Hv50k-2016-383164	Chr6	35934806	A/G	0.16	5.21	0.33
Salt-Na^+^	JHI-Hv50k-2016-272613	Chr4	639754617	T/C	0.25	4.40	0.14
	BOPA1_1954-1198	Chr4	639755186	T/C	0.25	4.40	0.14
	JHI-Hv50k-2016-272632	Chr4	639940903	T/C	0.31	4.52	0.14
	JHI-Hv50k-2016-272635	Chr4	639941872	A/G	0.26	4.84	0.15
	JHI-Hv50k-2016-272653	Chr4	639947136	A/G	0.27	6.55	0.18
	BOPA2_12_31219	Chr4	639950923	A/G	0.31	4.75	0.15
	JHI-Hv50k-2016-272665	Chr4	639953112	A/G	0.26	4.84	0.15
	JHI-Hv50k-2016-272672	Chr4	639956210	A/G	0.26	4.84	0.15
	SCRI_RS_222098	Chr4	639958857	T/C	0.31	4.75	0.15
	JHI-Hv50k-2016-272681	Chr4	639960860	G/C	0.26	4.84	0.15
	JHI-Hv50k-2016-272718	Chr4	640199193	T/C	0.26	4.84	0.15
	JHI-Hv50k-2016-272744	Chr4	640208490	A/G	0.30	4.83	0.15
	JHI-Hv50k-2016-497584	Chr7	608811182	G/C	0.15	6.30	0.17
	JHI-Hv50k-2016-497596	Chr7	608825674	A/G	0.14	6.09	0.17
	JHI-Hv50k-2016-497604	Chr7	608826566	T/A	0.15	6.30	0.17
	JHI-Hv50k-2016-497605	Chr7	608826839	T/C	0.14	6.09	0.17
	JHI-Hv50k-2016-497608	Chr7	608827049	A/G	0.14	6.09	0.17
	JHI-Hv50k-2016-497611	Chr7	608827306	T/A	0.15	6.30	0.17
	JHI-Hv50k-2016-497612	Chr7	608827493	C/G	0.07	6.37	0.17
	JHI-Hv50k-2016-497619	Chr7	608828515	T/C	0.14	6.09	0.17
	JHI-Hv50k-2016-497620	Chr7	608828546	T/C	0.14	6.09	0.17
Salt-Na^+^/K^+^	JHI-Hv50k-2016-272270	Chr4	638202451	A/G	0.29	4.77	0.18
	JHI-Hv50k-2016-272281	Chr4	638211825	C/G	0.29	4.86	0.19
	JHI-Hv50k-2016-272322	Chr4	638388495	A/G	0.29	4.93	0.19
	JHI-Hv50k-2016-272323	Chr4	638388551	A/G	0.29	4.93	0.19
	JHI-Hv50k-2016-272456	Chr4	638775104	C/G	0.22	4.85	0.19
	JHI-Hv50k-2016-272457	Chr4	638775254	A/C	0.23	4.50	0.18
	JHI-Hv50k-2016-272613	Chr4	639754617	T/C	0.25	5.02	0.19
	BOPA1_1954-1198	Chr4	639755186	T/C	0.25	5.02	0.19
	JHI-Hv50k-2016-272632	Chr4	639940903	T/C	0.31	4.42	0.18
	JHI-Hv50k-2016-272635	Chr4	639941872	A/G	0.26	5.78	0.20
	JHI-Hv50k-2016-272653	Chr4	639947136	A/G	0.27	7.35	0.23
	BOPA2_12_31219	Chr4	639950923	A/G	0.31	4.83	0.19
	JHI-Hv50k-2016-272665	Chr4	639953112	A/G	0.26	5.78	0.20
	JHI-Hv50k-2016-272672	Chr4	639956210	A/G	0.26	5.78	0.20
	SCRI_RS_222098	Chr4	639958857	T/C	0.31	4.83	0.19
	JHI-Hv50k-2016-272681	Chr4	639960860	G/C	0.26	5.78	0.20
	JHI-Hv50k-2016-272718	Chr4	640199193	T/C	0.26	5.78	0.20
	JHI-Hv50k-2016-272744	Chr4	640208490	A/G	0.30	5.10	0.19
	SCRI_RS_2513	Chr4	640506186	T/C	0.21	4.49	0.18
	JHI-Hv50k-2016-497584	Chr7	608811182	G/C	0.15	5.52	0.20
	JHI-Hv50k-2016-497596	Chr7	608825674	A/G	0.14	5.24	0.19
	JHI-Hv50k-2016-497604	Chr7	608826566	T/A	0.15	5.52	0.20
	JHI-Hv50k-2016-497605	Chr7	608826839	T/C	0.14	5.24	0.19
	JHI-Hv50k-2016-497608	Chr7	608827049	A/G	0.14	5.24	0.19
	JHI-Hv50k-2016-497611	Chr7	608827306	T/A	0.15	5.52	0.20
	JHI-Hv50k-2016-497612	Chr7	608827493	C/G	0.07	5.67	0.20
	JHI-Hv50k-2016-497619	Chr7	608828515	T/C	0.14	5.24	0.19
	JHI-Hv50k-2016-497620	Chr7	608828546	T/C	0.14	5.24	0.19
	JHI-Hv50k-2016-497769	Chr7	609661600	A/G	0.12	4.44	0.18
	JHI-Hv50k-2016-497771	Chr7	609661683	T/A	0.12	4.44	0.18
	JHI-Hv50k-2016-498906	Chr7	613715990	A/G	0.11	5.69	0.20
R-Na^+^	JHI-Hv50k-2016-272613	Chr4	639754617	T/C	0.25	4.71	0.27
	BOPA1_1954-1198	Chr4	639755186	T/C	0.25	4.71	0.27
	JHI-Hv50k-2016-272635	Chr4	639941872	A/G	0.26	4.57	0.27
	JHI-Hv50k-2016-272653	Chr4	639947136	A/G	0.27	4.88	0.27
	JHI-Hv50k-2016-272665	Chr4	639953112	A/G	0.26	4.57	0.27
	JHI-Hv50k-2016-272672	Chr4	639956210	A/G	0.26	4.57	0.27
	JHI-Hv50k-2016-272681	Chr4	639960860	G/C	0.26	4.57	0.27
	JHI-Hv50k-2016-272718	Chr4	640199193	T/C	0.26	4.57	0.27
	JHI-Hv50k-2016-272759	Chr4	640312902	A/C	0.28	4.40	0.27
	JHI-Hv50k-2016-405836	Chr6	432135007	A/G	0.42	4.84	0.27
	JHI-Hv50k-2016-405852	Chr6	432316393	T/C	0.42	4.84	0.27
	JHI-Hv50k-2016-405919	Chr6	433298409	T/G	0.42	5.06	0.28
	JHI-Hv50k-2016-405927	Chr6	433612074	A/G	0.42	4.84	0.27
	JHI-Hv50k-2016-405934	Chr6	433712844	A/C	0.42	4.84	0.27
	JHI-Hv50k-2016-405948	Chr6	434910711	A/T	0.42	4.84	0.27
	JHI-Hv50k-2016-405999	Chr6	435230460	T/G	0.42	4.84	0.27
	JHI-Hv50k-2016-406007	Chr6	435260539	A/G	0.42	4.84	0.27
	JHI-Hv50k-2016-406088	Chr6	436330505	A/C	0.42	4.84	0.27
	JHI-Hv50k-2016-406253	Chr6	439399412	T/C	0.42	4.45	0.27
R-Na^+^/K^+^	JHI-Hv50k-2016-271592	Chr4	636431702	G/C	0.10	4.61	0.24
	JHI-Hv50k-2016-271593	Chr4	636431784	T/C	0.10	4.61	0.24
	JHI-Hv50k-2016-272281	Chr4	638211825	C/G	0.29	4.47	0.24
	JHI-Hv50k-2016-272322	Chr4	638388495	A/G	0.29	4.73	0.24
	JHI-Hv50k-2016-272323	Chr4	638388551	A/G	0.29	4.73	0.24
	JHI-Hv50k-2016-272528	Chr4	639216140	A/G	0.27	4.55	0.24
	JHI-Hv50k-2016-272613	Chr4	639754617	T/C	0.25	6.77	0.28
	BOPA1_1954-1198	Chr4	639755186	T/C	0.25	6.77	0.28
	JHI-Hv50k-2016-272632	Chr4	639940903	T/C	0.31	4.64	0.24
	JHI-Hv50k-2016-272635	Chr4	639941872	A/G	0.26	6.66	0.27
	JHI-Hv50k-2016-272653	Chr4	639947136	A/G	0.27	6.70	0.27
	BOPA2_12_31219	Chr4	639950923	A/G	0.31	4.55	0.24
	JHI-Hv50k-2016-272665	Chr4	639953112	A/G	0.26	6.66	0.27
	JHI-Hv50k-2016-272672	Chr4	639956210	A/G	0.26	6.66	0.27
	SCRI_RS_222098	Chr4	639958857	T/C	0.31	4.55	0.24
	JHI-Hv50k-2016-272681	Chr4	639960860	G/C	0.26	6.66	0.27
	JHI-Hv50k-2016-272699	Chr4	640119802	A/C	0.29	5.31	0.25
	JHI-Hv50k-2016-272718	Chr4	640199193	T/C	0.26	6.66	0.27
	JHI-Hv50k-2016-272744	Chr4	640208490	A/G	0.30	4.99	0.25
	JHI-Hv50k-2016-272747	Chr4	640310734	T/G	0.28	5.20	0.25
	JHI-Hv50k-2016-272759	Chr4	640312902	A/C	0.28	5.79	0.26
	JHI-Hv50k-2016-405919	Chr6	433298409	T/G	0.42	4.40	0.24

-Log_10_(P) value indicates the significance levels and R^2^ (%) indicates the percentage of phenotypic variation explained by each SNP; MAF, minor allele frequency.

Under control treatment, we detected 8 significant SNPs that correlated with ion-related traits (**Figure 3A**). 2 SNPs for K^+^ content (CK-K^+^) were detected on Chr 2 located at 6.193 and 647.542 Mb, respectively. There were 6 SNPs associated with Na^+^ content (CK-Na^+^), including 2 on Chr 5 and 4 on Chr 6, respectively. Under salt treatment, 20 significant SNPs were identified including 7 on Chr 4 and 9 on Chr 7 for Salt-Na^+^, which were nearby on their respective chromosomes (**Figure 3B**). A single SNP explained 14.85 to 17.81% of phenotypic variation. The strongest associated SNP, JHI-Hv50k-2016-272653 (-Log_10_ (*P*)=6.55) was found at 639.94 Mb on Chr 4 (**Table 4**). For Salt-Na^+^/K^+^, 30 significant SNPs was found on Chr 4 and 7 (**Figure 3B**), and a single SNP explained 17.85 to 27.73% of the phenotypic variation. Among these SNPs, 20 have been previously detected for Salt-Na^+^, including the strongest associated SNP JHI-Hv50k-2016-272653 (-Log_10_ (*P*)=7.35).

For the other two traits, R-Na^+^ and R-Na^+^/K^+^, 19 and 21 loci were detected, respectively. 10 SNPs for R-Na^+^ were found on Chr 6 (**Figure 3C**). 21 SNPs for R-Na^+^/K^+^ were found on Chr 4. Of these, JHI-Hv50k-2016-272613 and BOPA2_12_31219 were the most strongly associated SNP (-Log_10_ (*P*) = 6.77) located at 639.75 Mb. However, we did not find any SNP sites significantly associated with Na^+^/K^+^ ratio under control treatment (CK-Na^+^/K^+^), Salt-K^+^, and R-K^+^ (**Figure 3**). Overall, the 3 SNP peaks located on Chr 4, 6, and 7 were detected repeatedly, indicating that these regions may be the genomic hot spot related to salt tolerance in barley (**Table 4**).

### 3.5 Analysis of major SNP loci in SNP peak relevant to the ion-related traits

**Figure 4:**
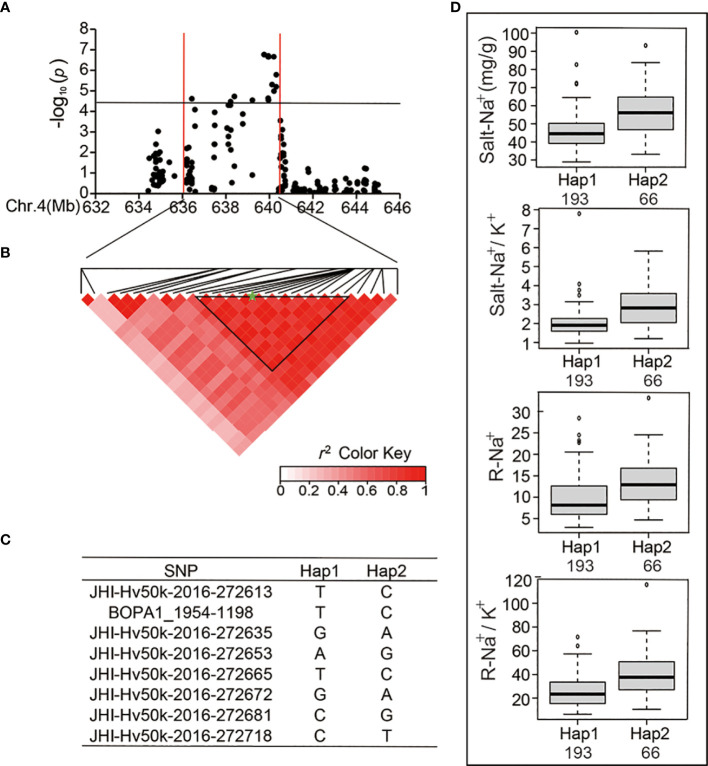
Analysis of the SNP peak and the candidate genes on chromosome 4. **(A)** Manhattan plots for Chr 4. The black line represents the significance threshold (*P* < 10^-4.40^) and a red line indicates the position of the strong SNP peak. **(B)** LD based on pairwise *r* ^2^ values between the SNPs estimated on Chr 4. The black inverted triangles indicate 8 significantly associated SNPs that were repeatedly detected. The green five-pointed star indicates the strongest SNP with the highest threshold. **(C)** Haplotypes were found among the barley accessions using the 8 SNPs. **(D)** Phenotypic differences of Salt-Na^+^, Salt-Na^+^/K^+^, R-Na^+^, and R-Na^+^/K^+^ between the two haplotypes.

Next, we analyzed major SNP loci that are relevant to the Salt-Na^+^, Salt-Na^+^/K^+^, R-Na^+^, and R-Na^+^/K^+^. We found 25 significant SNPs within a significant peak located at the end of chromosome 4 (**Table 4** and **Figure 4A**), which was detected in the previous studies (Hazzouri et al., 2018). There were eight SNPs detected repeatedly to further investigate the allelic variation. Haplotype analysis showed a high level of LD (*r* ^2^ = 0.77–1.0) between the eight associated SNPs (**Figure 4B**), which resulted in two haplotypes (**Figure 4C**). The average Salt-Na^+^ of Hap1 (45.58 mg/g) was less than that of Hap2 (**Figure 4D**). Similar to Salt-Na^+^ data, the average values of Salt-Na^+^/K^+^, R-Na^+,^ and R-Na^+^/K^+^ for Hap1 were 1.99, 9.58, and 25.51, respectively, which were all lower than that of Hap2 (**Figure 4D**).

**Figure 5:**
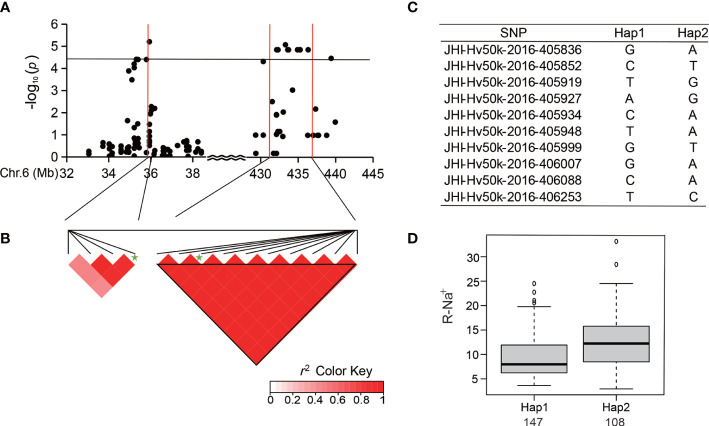
Analysis of the SNP peak on chromosome 6. **(A)** Manhattan plots for Chr 6. The black line represents the significance threshold (*P* < 10^-4.40^) and a red line indicates the position of the strongest SNP peak. **(B)** LD based on pairwise *r* ^2^ values between the SNPs estimated on Chr 6. The black inverted triangles show 10 significantly associated SNPs. The green five-pointed star indicates the strongest SNP with the highest threshold. **(C)** Haplotypes were observed in the barley accessions using the 10 SNPs. **(D)** Phenotypic differences of R-Na^+^ between the two haplotypes.

**Figure 6:**
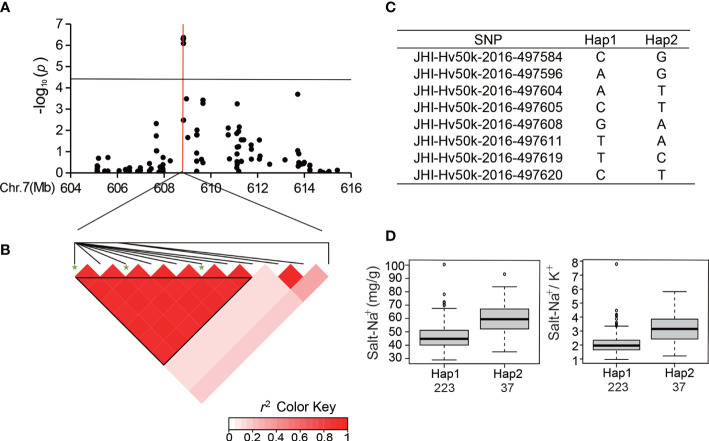
Analysis of the SNP peak on chromosome 7. **(A)** Manhattan plots for Chr 7. The black line represents the significance threshold (*P* < 10^-4.40^) and a red line indicates the position of the strongest SNP peak. **(B)** LD based on pairwise *r* ^2^ values between the SNPs estimated on Chr 7. The black inverted triangles show 8 significantly associated SNPs that were detected repeatedly. The green five-pointed star indicates the strongest SNP with the highest threshold. **(C)** Haplotypes were observed in the barley accessions using the 8 SNPs. **(D)** Phenotypic differences of Salt-Na^+^ and Salt-Na^+^/K^+^ between the two haplotypes.

Ten significant SNPs with a high level of LD located between 432 and 435 Mb at the end of Chr 6 were analyzed for R-Na^+^ (**Table 3** **;** **Figures 5A, B**). Among the 288 accessions, there were two haplotypes with the ten SNPs (**Figure 5C**), and the average R-Na^+^ of Hap1 (9.32) was 26.21% lower than that of Hap2 (**Figure 5D**). There was an SNP peak located at 608.8 Mb on Chr 7 (**Figure 6A**) containing 8 SNPs that were detected repeatedly and were in proximity related to Salt-Na^+^ and Salt-Na^+^/K^+^ traits. Haplotype analysis showed two haplotypes with a high level of LD (**Figures 6B, C**). The average Salt-Na^+^ and Salt-Na^+^/K^+^ of Hap1 were 46.46 mg/g and 2.07, respectively, which was significantly lower than the other one (**Figure 6D**).

For the genetic effect of each SNP in Hap1 of Chr4, for example, JHI-Hv50k-2016-272613 could be classified into three genotypes. And the TT genotype displayed a lower average Salt-Na^+^, Salt-Na^+^/K^+^, R-Na^+^, and R-Na^+^/K^+^, respectively, than the other nonincluded genotypes (**Supplementary Figure 2**). The other SNPs in Hap1 of Chr6 and Chr7 had the same effect on the phenotype as the locus JHI-Hv50k-2016-272613 with the lower Salt-Na^+^ and Salt-Na^+^/K^+^, respectively (**Supplementary Figure 3**) and R-Na^+^ (**Supplementary Figure 4**).

### 3.6 Exploration of candidate genes associated with SNPs

Based on the criteria of 500 Kb distance from significant SNPs, we found 616 candidate genes. More than half of these genes have no functional annotation. The rest of the annotated genes are involved in binding proteins, transport proteins, protein kinases, and other unknown function proteins (**Supplementary Table 5**). For example, *HORVU4Hr1G087960* code for a high-affinity K^+^ transporter (HvHKT1;5), which is involved in the ionic stress signaling pathway (Qiu et al., 2011; Hazzouri et al., 2018; van Bezouw et al., 2019; Huang et al., 2020; Houston et al., 2020; Wege et al., 2021). *HORVU5Hr1G124800* is annotated for serine/threonine-protein kinase and may be involved in MAPK cascades responding to salt or osmotic stress. Furthermore, GO enrichment and KEGG pathway analysis were carried out to explore the role of these genes.

GO analysis indicated that in terms of molecular function, the rRNA N-glycosylase activity (GO:0030598) and RNA glycosylase activity (GO:0030597) with the common four genes were the most significantly enriched categories (**Supplementary Table 6**). GO cellular component analysis suggested that the candidate genes are mainly located in the NADH dehydrogenase complex (GO:0030964), autophagosome membrane (GO:0000421), and mitochondrial membrane (GO:0031966). Meanwhile, 5 genes showed significant enrichment for biological processes such as negative regulation of translation (GO:0017148) and cellular amide metabolic process (GO:0034249). Likewise, KEGG pathway analysis revealed that only 5 genes were enriched in 9 pathways. *HORVU4Hr1G087100* encodes branched-chain-amino-acid aminotransferase 6, which participates in the synthesis of alanine, valine, leucine, and isoleucine. The other pathways include cytoskeletal regulation by Rho GTPase (*HORVU4Hr1G087740*), Huntington’s disease (*HORVU4Hr1G087740*, *HORVU4Hr1G088610*, and *HORVU6Hr1G016100*) and so on (**Supplementary Table 6**).

### 3.7 RNA-Seq analysis in response to salt stress in barley cultivars

To understand the regulatory mechanisms of salt stress and explore promising candidate genes, we determined the transcriptome changes in the leaves of CM72 and Gairdner under salt stress using RNA-seq. There were 21.54 million raw reads on average and more than 91.98% of the reads of each sample (0, 3, 12, and 48 h) aligned with the specified reference genome and led to the identification of 90,591 genes (**Supplementary Table 7**). With the increase of salt treatment time, the number of DEGs also gradually increased (**Supplementary Figure 5A**). Venn diagrams showing the number of distinct and common salt-responsive genes (DEGs) in the two contrasting genotypes in different point (**Supplementary Figure 5B**). Principal Component Analysis (PCA) showed that the samples separated into four major clusters, indicating that the salt treatment time was likely the largest source of variation in the data (**Supplementary Figure 5C**). Therefore, we chose the data of controls (0 h) and 48 h salt-treated samples of both genotypes to further bioinformatic analyses.

**Figure 7:**
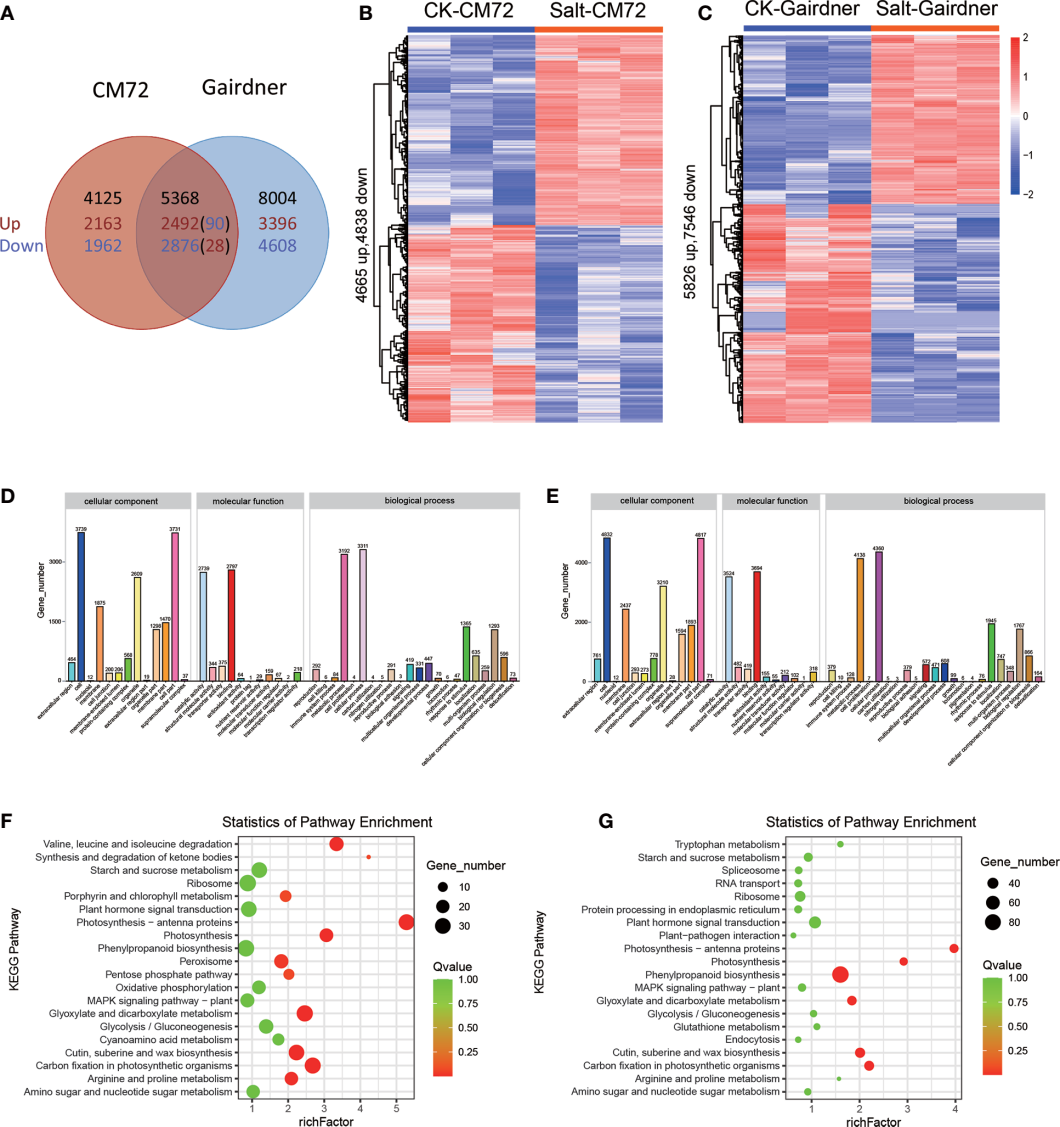
Transcriptome analysis in CM72 and Gairdner under salt conditions. **(A)** Venn diagram showing the number of transcripts detected as up and down-regulated (P < 0.01) in CM72 and Gairdner under salt conditions. **(B)** Heat maps showing differentially expressed transcripts in CM72 under control and salt conditions. **(C)** Heat maps showing differentially expressed transcripts in Gairdner under control and salt conditions. **(D)** The Gene Ontology (GO) enrichment analysis in CM72 under salt conditions. **(E)** The Gene Ontology (GO) enrichment analysis in Gairdner under salt conditions. **(F)** The top 20 significantly KEGG enrichment pathways in CM72 under salt conditions. **(G)** The top 20 significantly KEGG enrichment pathways in Gairdner under salt conditions.

After 48h of salt treatment, 17,437 genes were significantly differentially expressed under salt stress, and of these, 5368 genes were commonly deferentially expressed in both varieties, while 4,125 and 8,004 genes were specifically expressed in CM72 and Gairdner, respectively. Moreover, there were 90 up-regulated and 28 down-regulated genes in CM72 while which were down-regulated and up-regulated in Gairdner (**Figures 7A-C**).

Gene ontology (GO) enrichment analysis is performed to determine the potential function of DEGs in response to salt stress. The results of GO enrichment analysis indicated that in terms of cellular component the enriched same GO terms were cell, cell part, membrane, organelle in both CM72 and Gairdner. GO molecular function analysis suggested that the DEGs mainly richer in binding, catalytic activity, structural molecule activity, transporter activity in both CM72 and Gairdner. Within the biological process, the mainly enriched GO terms were biological regulation, cellular process, metabolic process, response to stimulus) in both CM72 and Gairdner (**Figures 7D, E**).

KEGG pathway enrichment analysis showed that the DEGs were mainly enriched into the pathway of “ribosome”, “phenylpropanoid biosynthesis” and “carbon fixation in photosynthetic organisms” in both varieties. Otherwise, the specific term KEGG pathway were “glyoxylate and dicarboxylate metabolism” and “photosynthesis - antenna proteins” in CM72, and “cutin, suberine and wax biosynthesis” and “plant hormone signal transduction” in Gairdner, respectively (**Figures 7F, G**).

### 3.8 Putative candidate genes identified by GWAS and RNA-seq analysis

**Figure 8:**
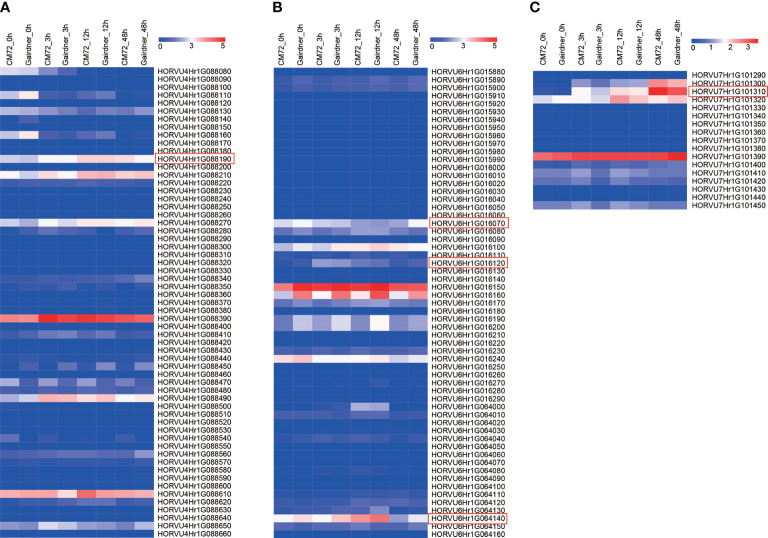
Expression pattern of candidate genes in the peak regions of Chr4, Chr6 and Chr7. **(A)** Heat map showing the expression levels of 59 candidate genes in Chr4 among two barley cultivars. **(B)** Heat map showing the expression levels of 59 candidate genes in Chr6 among two barley cultivars. **(C)** Heat map showing the expression levels of 17 candidate genes in Chr6 among two barley cultivars. The barley leaf samples were collected at 0, 3, 12, and 48 h under salt stress.

**Figure 9:**
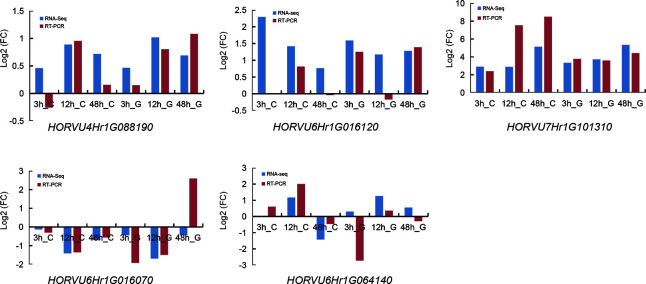
Expression pattern validation of five selected genes **(***HORVU4Hr1G088190* (PGK2), *HORVU6Hr1G016070* (BASS3), *HORVU6Hr1G016120* (SINAT2), *HORVU6Hr1G064140* (AQP), and *HORVU7Hr1G101310* (SYT3)), which were also detected by RNA-Seq, in CM72 and Gairdner leaves by qRT-PCR. The samples were collected at 0, 3, 12, and 48 h under salt stress.

Further, we investigated the expression level of the 616 genes and found that most of these showed no expression at the four-time points (**Supplementary Table 5**). A total of 59, 59, and 17 candidate genes were within the LD decay region (hot spots) on Chr 4, 6, and 7, respectively (**Figure 8** **;** **Supplementary Table 5**). Based on GWAS and RNA-seq analysis, we selected 5 candidate genes that are associated with the regulation of Na^+^/K^+^ balance (**Figure 9** and **Table 5**). Among them, *HORVU4Hr1G088190* is located in the SNPs peak with three significant SNPs JHI-Hv50k-2016-272632, JHI-Hv50k-2016-272635, and JHI-Hv50k-2016-272653 are at 639.7-639.95 Mb (**Figure 8A**). *HORVU6Hr1G016070* and *HORVU6Hr1G016120* are in proximity of peak SNP JHI-Hv50k-2016-383164 at 35.79-36.06 Mb. *HORVU6Hr1G064140* located near the peak SNP JHI-Hv50k-2016-405919 at 433.30-433.71 Mb (**Figure 8B**). These three genes were annotated as sodium bile acid symporter family protein, SINA (seven in absentia) E3 ubiquitin-protein ligase SINAT2, and aquaporin-like superfamily protein, respectively. *HORVU7Hr1G101310*, located within the SNPs peak JHI-Hv50k-2016-497584, JHI-Hv50k-2016-497604, and JHI-Hv50k-2016-497611 at 608.81 Mb (**Figure 8C**), encodes a calcium-dependent lipid-binding (CaLB domain) family protein. The other three selected genes, *HORVU2Hr1G002690*, *HORVU5Hr1G124800*, and *HORVU5Hr1G124880*, are located near the association peak SNP loci of other chromosomes for CK-Na^+^ and CK-K^+^. In addition, analysis of the eight candidate genes by qRT-PCR appeared to follow a similar pattern as RNA-Seq (**Figure 9**; **Supplementary Figure 6A**).

**Table 5:** The putative candidate genes in SNP peaks associated with salt-tolerance related traits identified by GWAS and RNA-seq.

Traits	Gene ID	Annotation	Distance to nearest associated SNP (Kb)	Regulation
Salt-Na+ Salt-Na^+^/K^+^R-Na^+^ R-Na^+^/K^+^	HORVU4Hr1G088190	2-phosphoglycerate kinase (PGK2)	0-100	–
R-Na^+^	HORVU6Hr1G064140	Aquaporin-like superfamily protein (AQP)	0-100	down
CK-Na^+^	HORVU6Hr1G016120	E3 ubiquitin-protein ligase SINAT2 (SINAT2)	0-100	up
CK-Na^+^	HORVU6Hr1G016070	Sodium Bile acid symporter family (BASS3)	0-100	down
Salt-Na^+^ Salt-Na^+^/K^+^	HORVU7Hr1G101310	Calcium-dependent lipid-binding (CaLB domain) family protein (SYT3)	0-100	up

CK-Na^+^, Na^+^ content in control treatment; Salt-Na^+^, Na^+^ content in salt treatment (300 mM NaCl); Salt-Na^+^/K^+^, Na^+^/K^+^ ratio in salt treatment (300 mM NaCl); R-Na^+^, Relative value of Na^+^; R-Na^+^/K^+^, Relative value of Na^+^/K^+^ ratio. Up and down represent up- or down-regulated DEGs after salt stress. “-” represent not significantly regulated after salt stress.

**Figure 10:**
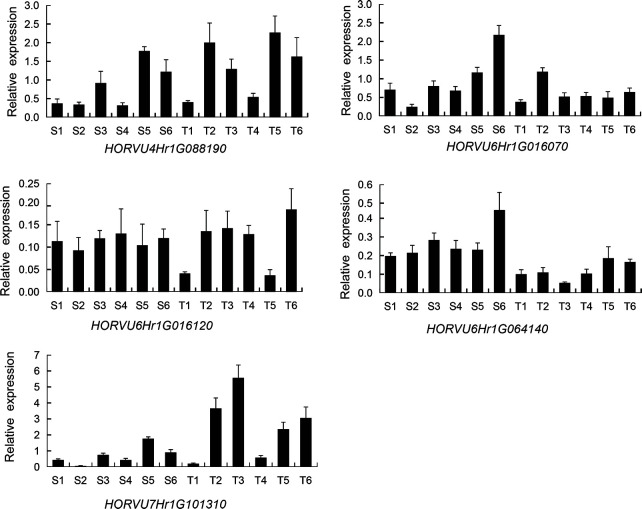
Relative expression of 5 candidate genes by RT-PCR in six salt-sensitive and six salt-tolerant varieties. *HORVU4Hr1G088190* (PGK2). *HORVU6Hr1G016070* (BASS3). *HORVU6Hr1G016120* (SINAT2). *HORVU6Hr1G064140* (AQP). *HORVU7Hr1G101310* (SYT3). Six salt-sensitive varieties, S1, Shan 3; S2, AKHELOL 1; S3, Boyer; S4, Rainbow; S5, Birka; S6, Gairdner; six salt-tolerant varieties; T1, PALLIDUM 043; T2, KIMALUNG; T3, Zhu Damai 5; T4, GURZAN; T5, Kunlun 14; T6, CM72.

The expression levels of the above 5 genes in the SNP peak region and other 3 genes were measured between the six salt-tolerant and six salt-sensitive varieties by qRT-PCR (**Figure 10**; **Supplementary Figure 6B**). *HORVU4Hr1G088190*, *HORVU7Hr1G101310*, and *HORVU5Hr1G124880* were up-regulated in the salt-tolerant varieties, while *HORVU6Hr1G016070*, *HORVU6Hr1G064140*, and *HORVU2Hr1G002690* were higher in the salt-sensitive varieties. This is consistent with the RNA-seq results showing that *HORVU7Hr1G101310* and *HORVU5Hr1G124880* were up-regulated and *HORVU6Hr1G016070, HORVU6Hr1G064140* were down-regulated (**Supplementary Table 5**). However, the DEGs *HORVU6Hr1G016120* and *HORVU5Hr1G124800* showed no difference in their expression level between the salt-sensitive and salt-tolerant varieties. In all, our data suggest that the above genes could be promising candidates genes as their orthologs also play a role in salt tolerance in other species (Hu et al., 2012; Liu et al., 2013; Joshi et al., 2016; Lee et al., 2019, Krausko et al., 2022, Myo et al., 2020, Wang et al., 2020).

## 4 Discussion

Increasing soil salinization has become a serious environmental challenge affecting crop yield and quality. Therefore, screening salt-tolerant crop varieties, identifying salt-tolerant genes, and breeding salt-tolerant varieties can be the most economical and effective strategies to alleviate the problem of soil salinization. The seedling stage which is most sensitive to stress is very important in the barley growth period. Therefore, the salt tolerance of barley measured by morphological and ion traits at the seedling stage reflects the salt tolerance of the variety to a certain extent. In our study, by phenotyping the 288 barley accessions under salt treatment, relatively abundant variation and significant differences among genotypes were observed for the ion traits that were selected to evaluate the salt tolerance of barley seedlings (**Supplementary Table 2**). Here, we identified some salt-tolerant varieties of barley with lower Na^+^ accumulation, R-Na^+^, Salt-Na^+^/K^+^, and Salt-Na^+^/K^+^ in leaves compared with salt-sensitive varieties (**Figure 1A** **;** **Supplementary Table 9**). Notably, previous studies have shown that salt-tolerant lines had higher Na^+^ content in roots (Tu et al., 2021), indicating the difference in translocation of Na^+^ from root to shoot among the two genotypes. Interestingly, the salt-tolerant varieties contain excellent Hap1 SNPs, while salt-sensitive varieties contain Hap2 SNPs. These findings can be further used to study the mechanism of salt tolerance in barley and prepare parents for salt tolerance breeding.

Some studies used QTL mapping to identify candidate intervals and molecular markers of salt tolerance in barley and the population constructed by bio-parents populations. Many QTL loci were identified (Mano and Takeda, 1997; Huang et al., 2009; Shavrukov et al., 2010; Angessa et al., 2017; Liu et al., 2017; Xue et al., 2017), but specific candidate genes related to salt tolerance could not be identified that slow the progress of salt-tolerant breeding in barley. GWAS, a novel and effective method, has been widely used to determine the genetic basis of crop plants. In recent years, several efforts have been made to detect QTLs related to salt tolerance using GWAS with simple sequence repeat (SSR) or SNP markers in barley (Long et al., 2013; Sbei et al., 2014; Elakhdar et al., 2016; Fan et al., 2016; Tu et al., 2021). However, the number of SNP markers was small which limited the mapping resolution and genome coverage, and also the SNPs were not found relevant to salt-tolerant loci on other chromosomes.

Here, we conducted a GWAS of ion-related salt-tolerant traits at the seedling stage of barley accessions having rich genetic diversity and 25,342 high-quality SNPs from the BarleySNP50K array for the first time. In total, 54 significant SNPs (*P* < 10^-4.40^) were identified for ion traits, and there were three SNP peaks located on Chr 4, 6, and 7 (**Figure 3** and **Table 3**). Furthermore, the SNP peak located on Chr 4 was consistent with the genetic interval noticed by Hazzouri et al. (2018) (**Figure 4**). Also, we found that the SNPs in the three SNP peaks can produce two haplotypes. Most of the barley germplasms belonged to the Hap1 group having lower Salt-Na^+^ content, R-Na^+^, Salt-Na^+^/K^+^ ratio, and/or R-Na^+^/K^+^ ratio (**Figures 4C, D**, **5C, D** and **6C, D**). This indicated that the Hap1 group has superior salt tolerance and may provide new insights into the genetic basis of salt tolerance in barley. In addition, the genetic effect of each SNP located in candidate genes, such as i.e.PGK2 with 3 significant SNPs (JHI-Hv50k-2016-272632, JHI-Hv50k-2016-272635, and JHI-Hv50k-2016-272653) (**Supplementary Figure 2**) on salt tolerance was manifested in that the genotypes in haplotype 1 reduced value of ion traits (**Supplementary Figures 3, 4**), and these SNPs could be used to develop functional molecular markers for further screening and identification of barley salt-tolerant germplasm from ion traits.

Meanwhile, we screened 616 candidate genes surrounding the significant SNPs that are involved in kinases, transcription factors, transporters, and cellular protein components (**Supplementary Table 5**). To further select possible salt tolerance genes, we performed transcriptome analysis in CM72 and Gairdner leaves. We found that the number of DEGs increased with salt treatment time, mainly enriched into the metabolic pathway, biosynthesis of secondary metabolites, photosynthesis, signal transduction and other pathways (**Figures 7F, G**), indicating that NaCl stress can promote the up-regulation or down-regulation of related genes *in vivo*, and adjust related pathways by changing the expression of genes to adapt to salt stress. Taken together, 8 promising salt-responding genes (PGK2, BASS3, SINAT2, AQP, SYT3, receptor kinase 2, GsSRK, and MATE) from the hot spot regions as protein kinases and transporters were identified by GWAS and RNA-Seq integrating analysis (**Figures 9**, **10**; **Supplementary Figure 5**), which might play roles in controlling ion content under salt stress. In addition, a total of 119 SNPs were found in the 8 putative candidate genes for alignment with the Morex genome in CM72 and Gairdner by RNA-seq data (**Supplementary Table 10**), showed the allelic variation among contrasting genotypes. However, whether these allelic variants could explain the salt tolerance of barley needs further study.

In previous studies, many ion transporter genes have been reported, involving Na^+^ absorption in roots, root-to-shoot Na^+^ transportation, Na^+^ translocation to shoots, and the localization of Na^+^ in the vacuole regionalization (Bahieldin et al., 2015; Gharaghanipor et al., 2022; Ouertani et al., 2022). In the current study, we have identified sodium transporter (*HKT1*), which was a down-regulated gene by RNA-seq, consistent with the previous findings that *HKT* negatively regulates salt tolerance when HKT;5 was knockdown, Na^+^ translocation from roots to shoots decrease, and increases in K^+^/Na^+^ in barley (Huang et al., 2020). However, some other well-known Na^+^ transporters (i.e. *NHX*, and *SOS* family genes) were not identified as common candidates (**Supplementary Table 5**), but we found that some novel genes in barley salt tolerance have not been reported and remain to be further verified and discussed in future research.

More importantly, some novel genes, including ion transporters and signal regulatory genes, were widely identified in the leaves of barley collections in salt response (**Figure 8**). *HORVU4Hr1G088190* encodes 2-phosphoglycerate kinase (PGK2), which is an important enzyme localized in cytoplasm as well as stroma of chloroplast in photosynthetic carbon metabolism, glycolysis, gluconeogenesis, and reductive pentose phosphate cycle (McMorrow and Bradbeer, 1990). It was demonstrated that ectopic expression of *OsPGK2-P* improved salt tolerance in tobacco by maintaining better ion homeostasis, and higher chlorophyll retention and proline accumulation (Kumari et al., 2009; Joshi et al., 2016). Earlier studies reported that the PGK2 is an isozyme of the glycolytic pathway that provides ATP in plants, also known as ATPase with ATP binding molecular function (GO:0005524) (**Supplementary Table 6**), including vacuolar H^+^-ATPase (V-ATPase) to transport H^+^ in the cytoplasm to the vacuole, and plasma membrane ATPase (plasma membrane H^+^-ATPase, PM-ATPase) to transport the H^+^ in the cytoplasm to the outside of the cell to maintain cellular pH homeostasis (Sawyer et al., 2008 Danshina et al., 2010; Krebs et al., 2010). *PGK2* was supposed to regulate ion homeostasis and maintain the intracellular pH balance with a high expression level in salt-tolerant varieties, although which was not the DEG in RNA-Seq analysis (**Table 5**). Additionally, the three genes *HORVU4Hr1G087760*, *HORVU4Hr1G087960*, and *HORVU4Hr1G088140* have been identified previously (Hazzouri et al., 2018),encoding bifunctional inhibitor/lipid-transfer protein/seed storage 2S albumin superfamily protein, sodium transporter HKT1 and expansin B2, respectively. In all, our results indicate that there could be a hot spot of salt tolerance candidate genes at the end of Chr 4 located at 639.94 Mb.

Additionally, the three genes *HORVU6Hr1G016070*, *HORVU6Hr1G016120*, and *HORVU6Hr1G064140* are homologous genes of BASS3, E3 ubiquitin-protein ligase SINAT2, and aquaporin PIP1 in rice, respectively. BASS family proteins encode a class of sodium/solute symporters with Na^+^-coupled metabolite transport properties which is predicted a Na^+^-dependent transporter (Zhou et al., 2014), so it may have a potential role in the Na^+^ physiology in plant salt tolerance. In wheat, TaBASS2, a putative pyruvate transporter, acts as a positive regulator of salinity tolerance *via* modulation of ABI4 expression (Zhao et al., 2016). The over-expression of *GhBASS5* in Arabidopsis impairs salt tolerance by increasing Na^+^ loading and accumulation (Myo et al., 2020). SINA E3 ubiquitin ligase is a RING (really interesting new gene) type E3 ubiquitin ligase, which is involved in plant growth and development, response to stress, interactions between plants and other organisms, and autophagy in plant cells. In rice, OsDIS1 (Oryza sativa drought-induced SINA protein 1) inhibited the drought resistance of rice by interacting with OsSKIPa (a positive regulator of drought and salt stress), affecting the expression of downstream stress response genes to regulate the drought resistance (Ning et al., 2011a; Ning et al., 2011b). In Arabidopsis, AtSINA2 can interact with CDKG1 (cyclin-dependent protein kinase G1), which phosphorylates AtSINA2 and affects its stability, thereby regulating the plant response to ABA and osmotic stress in *Arabidopsis* (Bao et al., 2014; Chen et al., 2018). Aquaporin (AQPs) plays a major role in maintaining the water status and transport across the plant which has water channel activity (GO:0015250) and involves ion transmembrane transport (GO:0034220) (**Supplementary Table 6**), that is crucial for plants to combat salt stress (Kumari and Bhatla, 2021). TaAQP8 confers salt stress tolerance by retaining a high K^+^/Na^+^ ratio and Ca^2+^ content and enhancing the antioxidant system (Hu et al., 2012). Aquaporin OsPIP1;1 functions as an active water channel that promotes rice salt resistance and seed germination (Liu et al., 2013). In tobacco, ectopic expression of *SpAQP1* from halophyte *Sesuvium portulacastrum* in transgenic tobacco could increase salt tolerance by enhancing the anti-oxidative activity of plants (Chang et al., 2016).

On Chr 7, the gene *HORVU7Hr1G101310* encodes a calcium-dependent lipid-binding (CaLB domain) family protein with molecular function of metal ion binding (GO:0046872) that homologous to Arabidopsis synaptotagmin-3. In *Arabidopsis*, the stress-induced SYT3 is an endoplasmic reticulum-plasma membrane (ER-PM) tether that also participates in maintaining PM integrity along with SYT1 (Ruiz-Lopez et al., 2021). Under salt stress, loss of AtSYT1 function resulted in a decrease in photosynthetic efficiency accompanied by a decrease in chlorophyll and carotenoids and an increase in flavonol content in leaves, aggravating the adverse effects of salt stress (Krausko et al., 2022). However, analysis of the above genes in barley salt tolerance has not been reported.

In addition, the gene *HORVU2Hr1G002690* encodes receptor kinase 2 and plays a crucial role in plant responses to abscisic acid (ABA), salt, and drought stresses (Sun et al., 2013a; Shi et al., 2014). The gene *HORVU5Hr1G124800* encodes a serine/threonine-protein kinase, also called G-type lectin S-receptor-like kinase (GsSRK) with ATP binding (GO:0005524), is involved in ion homeostasis, ROS scavenging, and osmotic regulation, and have a dual role in regulating both plant architecture and salt stress responses (Sun et al., 2013b; Sun M. Z. et al., 2018). The gene *HORVU5Hr1G124880* encodes the MATE efflux family protein with antiporter activity (GO:0015297) which modulates ABA sensitivity and increases tolerance to drought, salt, and cold stress with high production of antioxidant enzymes and significantly reduced levels of oxidants in plants (Zhang et al., 2014; Lu et al., 2018; Lu et al., 2019). On the other hand, the two other genes, *HORVU7Hr1G101590* and *HORVU7Hr1G103510* encode Cytochrome P450 superfamily protein and transducin/WD40 repeat-like superfamily protein (WD40), respectively. Both of these gene products participate in response to salt stress (Mao et al., 2013, Sun Z et al., 2018, Sun et al., 2021; Xia et al., 2021; Xin et al., 2021). However, the detailed function of these genes concerning salt tolerance in barley remains to be investigated by gene-editing and gain-of-function-based genetic engineering.

## 5 Conclusions

In this study, we first genotyped 288 diverse accessions having rich genetic diversity using the BarleySNP50K array. In total, 54 SNPs were significantly associated with salt tolerance traits and 616 candidate genes were identified by GWAS. There were 3 SNP peaks located on Chr 4, 6, and 7 that are associated with two and more traits. Furthermore, using integrated GWAS, RNA-seq, and qPCR analysis, we screened 5 putative genes (PGK2, BASS3, SINAT2, AQP, and SYT3) that may be related to ion balance in leaves from phenotypic and haplotype screening. This study provides novel SNPs and candidate genes for the study of salt tolerance in barley. Our results provide a basis for breeding new salt-tolerant barley varieties with marker-assisted selection.

## Data availability statement

The data presented in the study are deposited in the SRA database at NCBI repository, accession number PRJNA866193 and PRJNA892763 for RNA-seq data; and European Variation Archive repository for SNP data, accession number PRJEB59041.

## Author contributions

TX and WY conceived and designed the idea. TX, SM, XZ, JD, YZ, and XY performed the experiments. TX and WY analyzed the data. TX wrote and revised the manuscript. SM, XZ, JD, YZ, XY, and WY critically revised the article. All authors contributed to the article and approved the submitted version.
