# Corrigendum: Integrated GWAS and transcriptomic analysis reveal the candidate salt-responding genes regulating Na^+^/K^+^ balance in barley (*Hordeum vulgare* L.)

**DOI:** 10.3389/fpls.2023.1329188

**Published:** 2023-11-29

**Authors:** Tingting Xu, Shan Meng, Xiaopin Zhu, Jiachun Di, Yin Zhu, Xin Yang, Wei Yan

**Affiliations:** Institute of Germplasm Resources and Biotechnology, Jiangsu Key Laboratory for Agrobiolog, Jiangsu Provincial Platform for Conservation and Utilization of Agricultural Germplasm, Jiangsu Academy of Agricultural Sciences, Nanjing, China

**Keywords:** barley, GWAS, RNA-Seq, salt tolerance, Na +/K + balance, candidate genes

In the published article, there was an error in **Figure 4**. The image in **Figure 6** was used as the image in **Figure 4**. The corrected **Figure 4** appears below.

**Figure 4:**
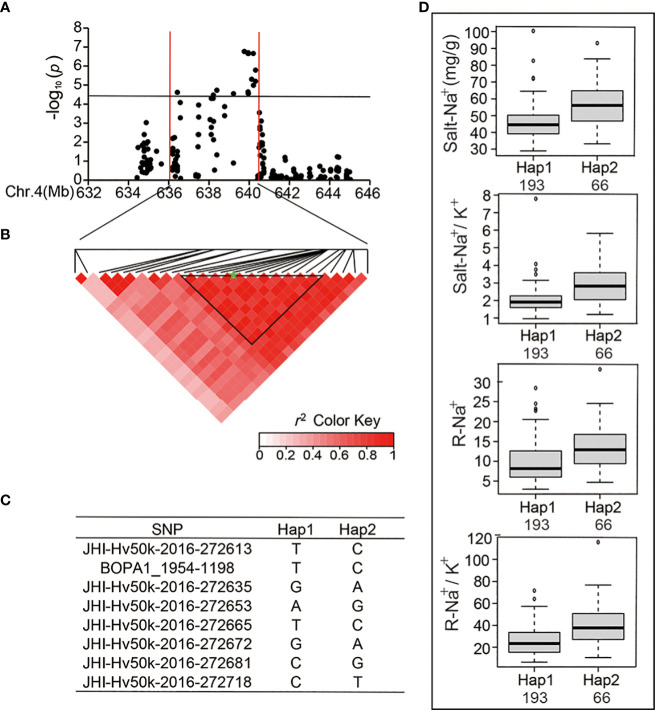
Analysis of the SNP peak and the candidate genes on chromosome 4. **(A)** Manhattan plots for Chr 4. The black line represents the significance threshold (*P* < 10^-4.40^) and a red line indicates the position of the strong SNP peak. **(B)** LD based on pairwise r2 values between the SNPs estimated on Chr 4. The black inverted triangles indicate 8 significantly associated SNPs that were repeatedly detected. The green five-pointed star indicates the strongest SNP with the highest threshold. **(C)** Haplotypes were found among the barley accessions using the 8 SNPs. **(D)** Phenotypic differences of Salt-Na^+^, Salt-Na^+^/K^+^, R-Na^+^, and R-Na^+^/K^+^ between the two haplotypes.

The authors apologize for this error and state that this does not change the scientific conclusions of the article in any way. The original article has been updated.

